# Photoprotective Effects of Two New Morin-Schiff Base Derivatives on UVB-Irradiated HaCaT Cells

**DOI:** 10.3390/antiox13010134

**Published:** 2024-01-22

**Authors:** Sara García-Gil, Azahara Rodríguez-Luna, Javier Ávila-Román, Gabriela Rodríguez-García, Rosa E. del Río, Virginia Motilva, Mario A. Gómez-Hurtado, Elena Talero

**Affiliations:** 1 Department of Pharmacology, Faculty of Pharmacy, Universidad de Sevilla, 41012 Seville, Spain; sgarcia18@us.es (S.G.-G.); motilva@us.es (V.M.); etalero@us.es (E.T.); 2 Faculty of Health Sciences, Universidad Loyola Andalucía, 41704 Seville, Spain; 3 Instituto de Investigaciones Químico Biológicas, Universidad Michoacana de San Nicolás de Hidalgo, Ciudad Universitaria, Morelia 58030, Michoacán, Mexico; gabriela.rodriguez@umich.mx (G.R.-G.); norma.del.rio@umich.mx (R.E.d.R.); mario.gomez@umich.mx (M.A.G.-H.)

**Keywords:** photoprotection, biological filter, UV protection, visprotection, morin, antioxidant, Schiff bases

## Abstract

Ultraviolet (UV) radiation harms the skin, causing oxidative damage, inflammation, and disruption of the skin’s barrier function. There is considerable interest in identifying new natural ingredients with antioxidant and anti-inflammatory properties to serve as adjuvants in sunscreens. The flavonoid morin (**1**) can undergo structural modifications to enhance its biological properties. The aim of this study was to synthesize two new morin-Schiff base derivatives, morin oxime (**2**) and morin semicarbazone (**3**), comparing their photoprotective effects with that of the parent compound on UVB-exposed HaCaT keratinocytes. The chemical structure of the novel compounds was revealed based on spectroscopic data analysis. Our findings demonstrated that derivatives **2** and **3** enhanced the light absorption capability in the UV–visible (vis) range compared to **1**. Tested compounds exhibited a higher scavenger capacity than Trolox. Moreover, pre-treatment with all compounds protected HaCaT cells from UVB-induced cell death. Compound **3** demonstrated the strongest antioxidant effect, reducing reactive oxygen species (ROS) generation and, subsequently, malondialdehyde (MDA) levels. Additionally, compounds **2** and **3** exhibited greater anti-inflammatory effects than compound **1**, significantly reducing interleukin (IL)-6 production levels at all tested concentrations. These findings have demonstrated, for the first time, a promising photoprotective activity of two new Schiff base derivatives and suggest their use as natural sunscreen ingredients.

## 1. Introduction

The skin acts as the initial protective barrier against a range of external factors, such as ultraviolet (UV) radiation, which causes oxidative harm to the skin, leading to inflammation and disruption of the skin’s barrier function. Extensive research confirms that exposure to UV radiation is the main contributor to the risk of developing skin cancer, accounting for approximately one-third of all cancer cases globally, as reported by the World Health Organization (WHO). Despite being highly preventable, the incidence of this type of cancer continues to increase [1].

Furthermore, UVB radiation is closely linked to skin aging, which is clinically characterized by dryness, pigmentation changes, loss of elasticity, wrinkles, and redness. Beyond photoaging, this radiation is involved in sunburn, photo-immunosuppression and skin cancer [2]. The sun exposure causes tissue damage, leading to functional and structural changes, accompanied by an underlying inflammatory and oxidative response that affects the components of the extracellular matrix and triggers the activation of proteolytic enzymes such as metalloproteinases, which can improve the proliferation of cancer cells [3,4].

Consequently, the use of sunscreens that effectively protect the skin from the harmful effects of solar radiation is of utmost importance. Currently, the cosmetics industry is making strides in developing new filters, and there is a growing emphasis on social awareness campaigns to prevent sun damage. However, factors such as incorrect or inadequate application of sunscreens, failure to reapply, or limited photostability of the filters do not ensure comprehensive sun protection, particularly in individuals with pre-existing dermatological conditions or high sensitivity to solar radiation [5].

Therefore, there is considerable interest in identifying new compounds with antioxidant and anti-inflammatory properties that can serve as adjuvants in sunscreens, enhancing their photoprotective effects. These compounds are not only intended to augment the sun protection factor (SPF) against UVB radiation, but also to offer specific protection against oxidative damage, immunosuppression, and pigmentation caused by sun exposure [6]. Furthermore, it is crucial that these protective compounds demonstrate safety not only for the body, but also for the environment [7]. Thus, the use of natural ingredients is increasing because they are more environmentally friendly and less toxic to the marine ecosystem [8].

In terms of photoprotective compounds, flavonols are a subclass of flavonoids that exhibit exceptional potential to protect the human body against reactive oxygen species (ROS). Among these flavonols, morin (**1**) emerges as a prominent candidate due to its low toxicity [9]. This flavonoid, alternatively referred to as 2′,3,4′,5,7-pentahydroxyflavone, represents a polyphenolic compound originally derived from plants within the Moraceae family such as the mulberry tree (*Maclura tinctoria*) and the Osage orange tree (*Maclura pomifera*). Moreover, it has been detected in species associated with the Rosaceae, Fagaceae, and Myrtaceae families, including the guajava tree (*Psidium guajava* L.) and *Ficus carica*, among others [10]. Like other polyphenols, which can protect against UV radiation by reducing free radicals, ROS, and pro-inflammatory cytokines [11], compound **1** has potent antioxidant properties [12]. It has been shown to exhibit several beneficial biological activities, including anticancer, photoprotective, antiaging, and neuroprotective effects, among others [13,14,15,16].

Like other polyphenols found in nature, compound **1** can undergo structural modifications to enhance its stability and adaptability for various applications. Chemical derivatization is a method used to improve the chemical stability and pharmacological properties of these compounds, which have potential therapeutic benefits [17]. Thus, in this study, a chemical modification of the compound **1** structure, which involves using its carbonyl group to prepare Schiff bases, has been proposed to improve the stability of the molecule. It is known that a Schiff base is a nitrogen analogue derived from an aldehyde or ketone, in which the carbonyl bond (C=O) is replaced by an imine (C=NR). They present extensive applications and have demonstrated a wide range of biological activities, including antifungal, antibacterial, antimalarial, antiproliferative, anti-inflammatory, antiviral, and antipyretic properties. The importance of the imine group in these compounds has been emphasized due to its crucial role in their biological activities [18].

It is important to note that, currently, no previous studies have reported any structural modifications of the phenolic compound **1** for the synthesis of Schiff bases, specifically with the aim of investigating their potential as photoprotective agents. Therefore, in this study, two new morin-Schiff base derivatives were synthesized, morin oxime (**2**) and morin semicarbazone (**3**), and their photoprotective effects were compared with those of the parent compound (**1**) by using an in vitro model of human keratinocytes (HaCaT) exposed to UVB radiation.

## 2. Materials and Methods

### 2.1. Reagents and Elucidation Instrumental

Morin hydrate (M4008), semicarbazide (363634), hydroxylamine hydrochloride (159417), and sodium acetate (236500) were acquired from Aldrich. (Sigma Aldrich, Saint Louis, MO, USA), Melting points were determined on a Fisher-Johns apparatus (Thermo Fisher Scientific Inc., Waltham, MA, USA) and were uncorrected. The ^1^H and ^13^C NMR spectra were measured at 400 MHz for ^1^H and at 100 MHz for ^13^C using a Varian Mercury 400 spectrometer manufactured by Varian, Inc. (Palo Alto, CA, USA) operating at a field of 9.4 Tesla from DMSO-_d6_ solutions using tetramethylsilane as the internal reference. Chemical shift values were reported in parts per million and coupling constants (*J*) in Hz. IR spectra were acquired on a Buck 500 spectrophotometer (Buck Scientific, Norwalk, CT, USA). HRMS spectra were acquired on a Bruker MicroTOF-II spectrometer (Bruker Corp., Billerica, MA, USA). Ultraviolet–visible (UV–Vis) spectra were measured on a Perkin Elmer Lambda (PerkinElmer, Inc., Waltham, Massachusetts, USA) spectrometer at 25 °C using EtOH solution. The molar absorption coefficient data (*ε* = A(*λ*)/cl) were given in log *ε* values.

### 2.2. General Procedure for Preparation of Morin-Schiff Derivatives

A proper batch of sodium acetate (20.6 mg for preparation of **2**, or 30.6 mg for yielding **3** and the respective Schiff base precursor hydroxylamine hydrochloride for preparation of **2** or semicarbazide for preparation of **3**) were mixed at the same weight proportion in a flask and dissolved with MeOH (3 mL). Next, morin hydrate (30 mg) previously dissolved in MeOH (2 mL) was added to the reaction mixture, which was stirred and heated for 3 h. Next, the respective crude reaction was poured on wet ice to yield a solid that was filtered and washed consecutively with dichloromethane and methanol.

***Morin oxime* (2)**. Yellow crystals (m.p. 291–293 °C); ^1^H NMR *δ* 13.24 (1H, s), 9.50 (1H, brs) 7.38 (1H, d, *J* = 9.0 Hz), 6.36 (1H, d, *J* = 2.0 Hz), 6.25 (1H, dd, *J* = 9.0, 2.0 Hz), 6.16 (1H, d, *J* = 2.0 Hz), 6.13 (1H, d *J* = 2.5 Hz). ^13^C NMR *δ* 178.0, 162.8, 161.7, 160.9, 160.3, 156.3, 149.0, 140.2, 128.5, 112.8, 105.6, 104.8, 103.5, 97.2, 92.9. UV (EtOH) λ_max_ (log *ε*) 210 (4.6), 265 (4.4), 390 (4.4). IR 3074, 1654, 1608, 1503, 1423, 1378, 1321, 1246, 1100. HRMS *m*/*z* 317.0579 [M]^+^ (calcd for C_15_H_11_NO_7_ 317.0536).

***Morin semicarbazone* (3)**. Brown crystals (m.p. 269–270 °C); ^1^H NMR *δ* 13.41 (1H, s), 9.37 (1H, brs), 7.40 (1H, d, *J* = 9.0 Hz), 6.32 (1H, d, *J* = 2.5 Hz), 6.20 (1H, dd, *J* = 9.0; 2.5 Hz), 6.08 (1H, d, *J* = 2.5 Hz), 6.06 (1H, d, *J* = 2.5 Hz). ^13^C NMR *δ* 178.5, 163.1, 162.5, 161.0, 160.2, 156.3, 149.0, 141.2, 127.8, 127.8, 113.7, 105.2, 105.2, 103.5, 97.0, 92.7. UV (EtOH) λ_max_ (log *ε*) 210 (4.5), 265 (4.2), 390 (4.1). IR 3335, 1651, 1608, 1574, 1505, 1378, 1308, 1224, 1173. HRMS *m*/*z* 359.0758 [M]^+^ (calcd for C_16_H_13_N_3_O_7_ 359.0753).

### 2.3. Cell Culture and UVB Irradiation of HaCaT Keratinocytes

Human immortalized keratinocytes, HaCaT, were obtained from CLS Cell Lines Service GmbH (Cytion, Eppelheim, Germany). Cells were maintained in high glucose Dulbecco’s modified Eagle medium (DMEM, GIBCO, Grand Island, NY, USA) containing 2 mM l-glutamine at 37 °C in a 5% CO_2_ incubator. The medium was supplemented with 10% heat-inactivated fetal bovine serum, 100 U/mL penicillin and 100 mg/mL streptomycin (BIOWEST, Nuaillé, France).

Concisely, the cells were cultured in 96-well plates (1.5 × 10^4^ cells/well) and incubated for 24 h at 37 °C and 5% CO_2_. Then, keratinocytes were exposed to **1** and derivatives **2** and **3** for 4 h. Following treatment, the culture medium was removed and replaced with a thin layer of PBS. Subsequently, to determine UVB exposure conditions, the cells were exposed to a single dose of UVB radiation (50 mJ/cm^2^) for different times (15, 30 and 60 s) or to different UVB doses (30, 50 and 100 mJ/cm^2^) using a Bio link cross-linker, VILBER (Collégien, France). This UV source emitted light within the energy spectrum of 280 to 315 nm, with a peak intensity at 302 nm, which corresponds to the UVB spectrum. Following UVB irradiation, the cells were provided with fresh complete medium and incubated for 24 h [19]. Then, the cell viability was examined to determine the experimental conditions for the following studies.

### 2.4. Assessment of Cell Viability by Resazurin Assay in HaCaT Keratinocytes

The effects of **1** and the two semisynthetic derivatives (**2** and **3**) on cell viability were determined using the resazurin technique. Resazurin is a blue dye that cells can reduce to resorufin. The quantity of resorufin produced is directly proportional to the number of viable cells. Firstly, to evaluate the possible cytotoxic effects of the compounds on HaCaT cells, they were seeded into 96-well plates (1.5 × 10^4^ cells/well) and incubated for 24 h at 37 °C and 5% CO_2_. Then, the keratinocytes were treated with different concentrations of the compounds (0.625, 1.25, 2.5, 5, and 10 µM) and incubated for another 24 h. To determine the photoprotective effect of the compounds, the cells were preincubated with the compounds at the same concentrations for 4 h. Subsequently, keratinocytes were washed with PBS and exposed to the selected UVB dose (50 mJ/cm^2^) for 30 s. PBS was replaced with fresh culture medium and incubated for 24 h. For the two experimental approaches, the medium was replaced and 200 µL of resazurin (20 µg/mL) were added for 3–4 h. The absorbance was measured at 540 and 620 nm using a spectrophotometer reader (iMark^TM^ microplate reader, BIO-RAD, Hercules, CA, USA) [20].

### 2.5. Analysis of Scavenger Activity by ABTS Test

The common free radical 2,2-azinobis(3-ethylbenzothiazoline-6-sulfonic acid) (ABTS) was used to determine the antioxidant scavenging activity of the compounds. The ABTS radical cation is generated by reacting the ABTS salt with a strong oxidizing agent, such as potassium persulfate. The reduction of the blue-green ABTS radical by hydrogen-donating antioxidants is measured through the suppression of its characteristic long-wavelength absorption spectrum. Thus, the ABTS solution was prepared and allowed to react in the dark overnight. Before being used, the ABTS solution was diluted with absolute ethanol. Then, 100 µL of different dilutions (3.12 to 200 µM) of **1** and the two semisynthetic derivates (**2** and **3**) were mixed with 100 µL of ABTS solution in a 96-well plate. Typically, the results are represented as the antioxidant capacity equivalent to Trolox, known as TEAC, and the absorbance was measured at 734 nm by using a spectrophotometer reader (iMark^TM^ microplate reader, BIO-RAD, Hercules, CA, USA) [21].

### 2.6. Evaluation of Intracellular ROS Production by DCF-DA Assay in UVB-Exposed HaCaT Keratinocytes

The 2′,7′-dichlorodihydrofluorescein diacetate (DCF-DA) assay Kit (Abcam^®^, Cambridge, UK) was used to determine intracellular ROS levels in UVB-exposed HaCaT keratinocytes [22]. Briefly, cells were seeded into 96-well black plates (2 × 10^4^ cells/well) and incubated for 24 h. HaCaT keratinocytes were pre-treated with **1** and the two semisynthetic derivates (**2** and **3**) at 2.5, 5, and 10 µM for 4 h. Treatments were replaced by a thin layer of PBS and exposed to UVB radiation (50 mJ/cm^2^). After irradiation, DCF-DA (20 µM) was added and incubated for 45 min, according to the manufacturer’s instructions. The fluorescence emitted by the 2′,7′-dichlorofluorescein (DCF) product was measured by using a fluorescence plate reader (Sinergy H1, Biotek^®^, Bad Friedrichshall, Germany) at 485 nm for excitation and 535 nm for emission.

### 2.7. Determination of Lipid Peroxidation by TBARS Assay in UVB-Exposed HaCaT Keratinocytes

The assessment of lipid peroxidation was conducted using the thiobarbituric acid reactive substance (TBARS) assay, following the protocol published by Iosageanu et al., with some modifications [23]. This method quantifies malondialdehyde (MDA), which is generated as a byproduct during the breakdown of endoperoxides in unsaturated fatty acids as a result of lipid substrate oxidation. Cells were seeded in 6-well plates at a density of 5 × 10^5^ cells/well and incubated for 24 h. HaCaT keratinocytes were pre-treated with **1** and the two semisynthetic derivates (**2** and **3**) at concentrations of 2.5, 5, and 10 µM for 4 h, followed by irradiation at 50 mJ/cm^2^. After 24 h of irradiation, cells were trypsinized, collected, centrifuged, and resuspended in a solution containing 0.5% thiobarbituric acid, prepared in 20% trichloroacetic acid. Samples were incubated for 30 min at 95 °C and subsequently centrifuged for 15 min at 10,000 rpm. Supernatants were collected to measure the absorbance at 540 nm using an iMark^TM^ microplate reader (BIO-RAD, Hercules, CA, USA). The levels of MDA were calculated and referred to the amount of protein (mg).

### 2.8. Determination of IL-6 Production by ELISA Kit in UVB-Exposed HaCaT Keratinocytes

HaCaT cells were seeded in 96-well plates (1.5 × 10^4^ cells/well) and incubated for 24 h at 37 °C. The cells were then pre-treated with morin (**1**) and the two semisynthetic derivatives (**2** and **3**) at 2.5, 5, and 10 µM for 4 h. After pre-treatment, the medium was removed and replaced by a thin layer of PBS and the cells were irradiated (50 mJ/cm^2^) for 30 s. Then, PBS was removed and replaced by fresh medium (100 µL) for 24 h. Next, the supernatants were collected and stored at −80 °C until use. A commercial ELISA kit (Diaclone GEN-PROBE, Besançon, France) was used to determine IL-6 levels according to the manufacturer’s instructions. The absorbance was measured at 450 nm in a microplate reader (iMarkTM microplate reader, BIO-RAD, Hercules, CA, USA). The results were expressed as IL-6 production relative to the percentage of viable cells due to the effect of UVB irradiation on cell viability.

### 2.9. Statistical Analysis

All values presented in the figures, tables, and text are expressed as arithmetic means ± standard error of the mean (SEM). Data were analyzed using GraphPad Prism, Version 5.00 (GraphPad Software, Inc., San Diego, CA, USA). The normality of the data was assessed using the Shapiro–Wilk normality test. The specific statistical tests employed for individual analyses are provided in the figure legends.

## 3. Results

### 3.1. Obtention of Morin Oxime (***2***) and Morin Semicarbazone (***3***)

Morin (**1**) reacted with hydroxylamine to provide morin oxime (**2**) in 52% yield as yellow crystals (m.p. 291–293 °C) whose HRMS showed *m*/*z* 317.0579 [M]^+^ (calcd for C_15_H_11_NO_7_ 317.0536). The IR spectrum highlighted a broad vibrational band at 3074 cm^−1^ (O-H) and the key vibration band at 1654 cm^−1^ from C=N moiety. The ^1^H NMR spectrum revealed the OH-5 proton at δ 13.24 and the typical flavonol skeleton resonances in the range of δ 7.38–6.13, of which H-6′ appeared at δ 7.38 (d, *J* = 9.0 Hz), H-3′ was observed as a doublet at δ 6.36 (*J* = 2.0 Hz), while H-5′ provided a double doublet at δ 6.25 (*J* = 9.0, 2.0 Hz). The ^1^H resonances of protons from Ring A appeared as doublets (*J* = 2.0 Hz) at δ 6.16 and δ 6.13. The ^13^C NMR showed fifteen signals in the range of δ 178–92.9, suggesting the formation of a new product. The spectroscopic data is available in the Supplementary Materials (Figures S1–S4). According to these results, the formation of **2** was established.

**Figure 1:**
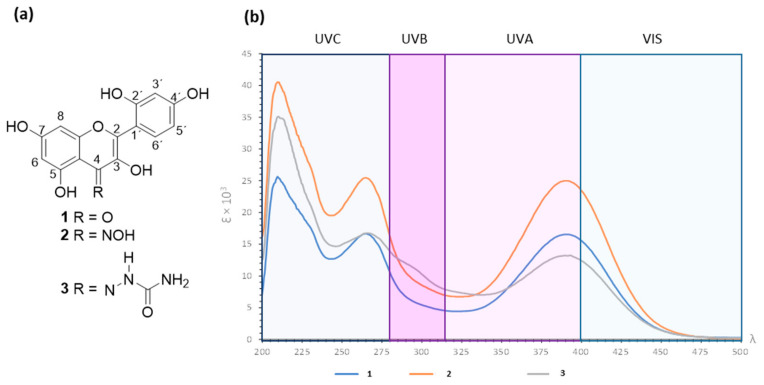
(**a**) Chemical structures of morin (**1**), morin oxime (**2**), and morin semicarbazone (**3**). (**b**) Absorbance spectra for compounds **1**–**3** in the UV–vis range.

On the other hand, the reaction of morin (**1**) with semicarbazide provided brown crystals (m.p. 268–270 °C) in 76% yield whose HRMS showed *m*/*z* 359.0758 [M]^+^ (calcd for C_16_H_13_N_3_O_7_ 359.0753). The IR spectrum revealed a broad vibrational band at 3335 cm^−1^ from N-H and O-H stretch vibrations, as well as C=N vibrations at 1651 and 1608 cm^−1^ from the semicarbazone motif. The ^1^H NMR spectrum showed a singlet at δ 13.41 from OH-5 proton; at δ 7.40 the H-6′resonance was revealed as doublet (9.0 Hz); the H-3′ proton appeared as doublet at δ 6.32 (2.5 Hz); the H-5′ resonance was observed as double doublet at δ 6.20 (9.0; 2.5 Hz); while H-6 and H-8 resonances appeared at δ 6.08 (*J* = 2.5 Hz) and 6.06 (*J* = 2.5 Hz), respectively. The ^13^C NMR revealed fourteen signals in the range of δ 178.5–92.7, suggesting the overlapping of several carbon resonances. Herein, C-4 was observed at δ 178.5 while carbonyl from semicarbazone moiety appeared at δ 160.2. These results suggested the formation of **3**. Formulas of **1** and its nitrogenated derivatives (**2** and **3**) are depicted in Figure 1a.

### 3.2. Absorbance Spectra for Morin (***1***) and Derivatives ***2*** and ***3*** in the UV–Vis Range

As experimental evidence of the enhanced photoprotective capacity of the newly synthesized compounds, a comparison of the UV–Vis absorption capacity was conducted between **1** and its nitrogenated derivatives (**2** and **3**). As depicted in Figure 1b, the UV–Vis absorption range encompasses a window spanning 200–500 nm, for the three compounds assayed, ensuring at least the absorption of UVB, UVA, and UVA–Vis rays, as they absorb above 400 nm. The tangible improvement through chemical modification for the synthesized compounds is evidenced by comparing the values of molar extinction coefficient (*ε*), which represents the ability of chemical species to absorb a specific wavelength of light. The results revealed that compounds **2** and **3** provided three maximum absorbances at λ_max_ 210, 265, and 390 nm. According to the Beer–Lambert law, compound **2** provided *ε* values of 40.426 (log *ε* 4.6), 25.450 (log *ε* 4.4), and 24.976 (log *ε* 4.4) at the respective λ_max_. Compound **3** showed *ε* values of 35.090 (log *ε* 4.5), 16.756 (log *ε* 4.2), and 13.198 (log *ε* 4.1), at the corresponding λ_max_. These *ε* results represented an improvement in UV–Vis absorption when compared to those data from **1** since *ε* from the parent compound were 25.453 (log *ε* 4.4; λ_max_ 210), 16.644 (log *ε* 4.2; λ_max_ 265), and 16.549 (log *ε* 4.2; λ_max_ 390). Thus, **2** exhibited the highest *ε* values, followed by **3**. Following a thorough analysis, it can be observed that the *ε*_max_ of **2** is 60% higher than that of **1** at λ_max_ 265 and 390 nm, and the *ε*_max_ of **3** is 40% higher than that of morin **1** at λ_max_ 210 nm (Table S1). Therefore, chemical modification led to an enhanced light absorption capability at the 200–500 nm wavelength for **2** and **3**, consequently elevating their effectiveness in comparison to the reference compound.

### 3.3. Photoprotective Effect of Morin (***1***) and Derivatives ***2*** and ***3*** on Cell Viability

**Figure 2:**
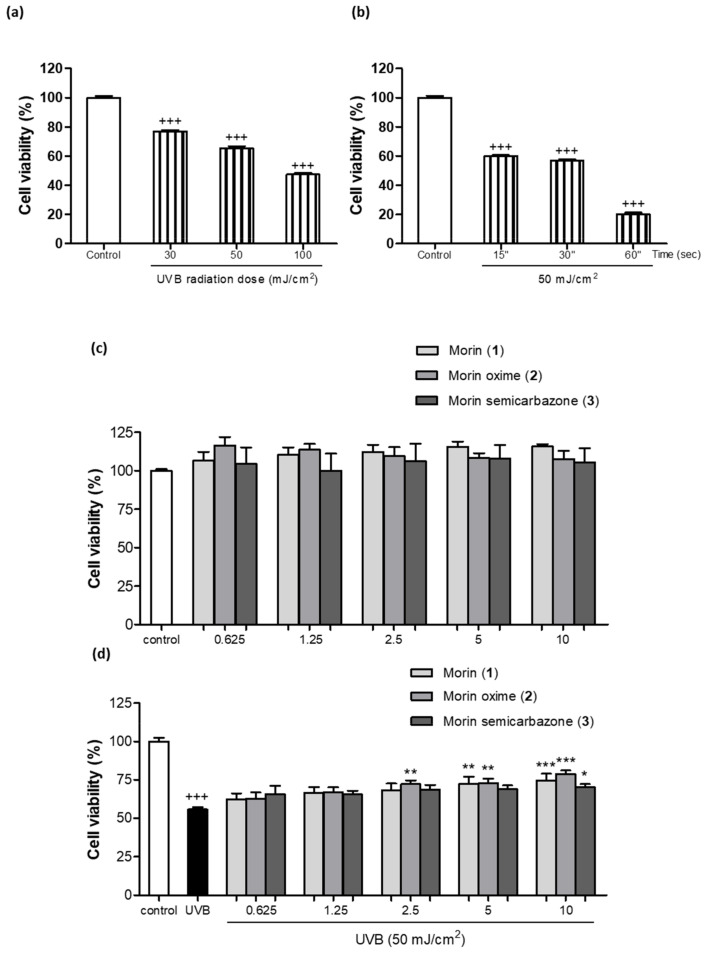
Photoprotective effect of morin (**1**) and its derivatives morin oxime (**2**) and morin semicarbazone (**3**) in UVB-irradiated keratinocytes. Resazurin assay results were represented as a percentage relative to untreated control cells to compare cell viability. (**a**) Effect of different UVB doses (30, 50, and 100 mJ/cm^2^) on cell viability. (**b**) Effect of the dose of 50 mJ/cm^2^ at different exposure times (15, 30, and 60 s) on cell viability. (**c**) Effects of **1** and its semisynthetic derivatives **2** and **3** on cell viability in non-exposed human HaCaT keratinocytes. Cells were pre-treated with the compounds at 0.625, 1.25, 2.5, 5, and 10 µM for 24 h. (**d**) Effects of pre-treatment with **1**, **2**, and **3** on cell viability in UVB-exposed HaCaT keratinocytes. Cells were pre-treated with the compounds at the same concentrations for 4 h, and then exposed to 50 mJ/cm^2^ for 30 s and incubated for 24 h. The results are presented as a percentage relative to untreated control cells, and the bars in the graph represent the mean ± standard error of the mean (SEM) values. The experiments were performed with a sample size of 6 (*n* = 6). The mean value was significantly different compared to control cells (+++ *p* < 0.001; Student’s *t*-test). The mean was also significantly different compared to the UVB-irradiated cells (* *p* < 0.05, ** *p* < 0.01, *** *p* < 0.001; one-way ANOVA followed by Bonferroni’s).

Cell viability 24 h after UVB exposure was determined using the resazurin assay. Since the objective to evaluate the photoprotective effect of these new compounds was to use a UVB dosage that would result in over 50% cell survival, different UVB doses (30, 50, and 100 mJ/cm^2^) were assayed (Figure 2a). In addition, the radiation dose of 50 mJ/cm^2^ was tested at different exposure times (15, 30, and 60 s) (Figure 2b). Finally, a dose of 50 mJ/cm^2^ for a duration of 30 s was selected for further irradiation experiments. As shown in Figure 2c, the compound **1** and the two semisynthetic derivatives (**2** and **3**) at concentrations ranging from 0.625 to 10 µM were incubated to assess their potential toxicity. The results from the resazurin assay indicated that none of the tested compounds at the evaluated doses exhibited toxicity towards the keratinocytes, demonstrating a favorable safety profile for these compounds. Therefore, all these concentrations were used to evaluate the preventive effect of these compounds against UVB irradiation. As shown in Figure 2d, exposure to UVB light resulted in a significant increase in cell mortality, affecting over 50% of the evaluated cells compared to the non-irradiated control cells. In contrast, the pre-treatment of cells with **1** (5 and 10 µM), **2** (2.5, 5, and 10 µM) and **3** at 10 µM prevented cell damage, which was evidenced by a significant increase in cell viability following UVB exposure.

### 3.4. Antioxidant Activity of Morin (***1***) and Derivatives ***2*** and ***3***

**Figure 3:**
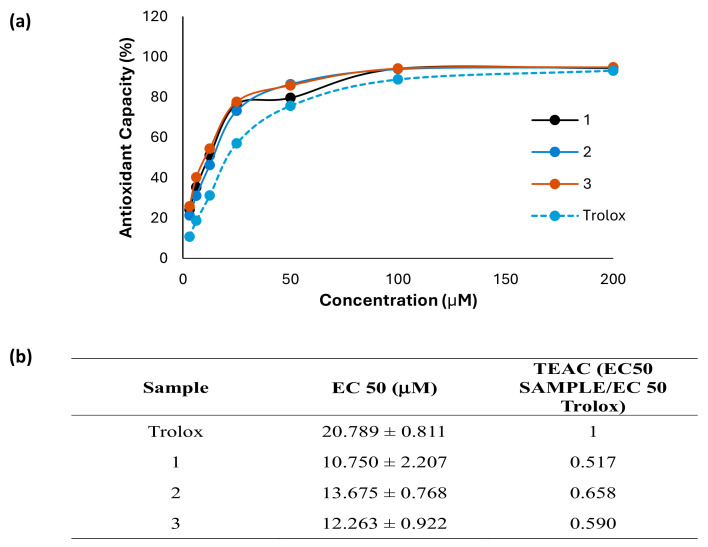
Antioxidant capacity of morin (**1**) and its derivatives morin oxime (**2**) and morin semicarbazone (**3**). (**a**) The free radical scavenger capacity was assessed at the range of 3.12–200 μM using the ABTS (2,2′-azino-bis (3-ethylbenzothiazoline-6-sulfonic acid)) test. (**b**) The half-effective concentration (EC50) was calculated, and the results were expressed as Trolox equivalent antioxidant capacity, TEAC (EC50 sample/EC50 Trolox). Results are representative of four independent experiments (*n* = 4).

**Figure 4:**
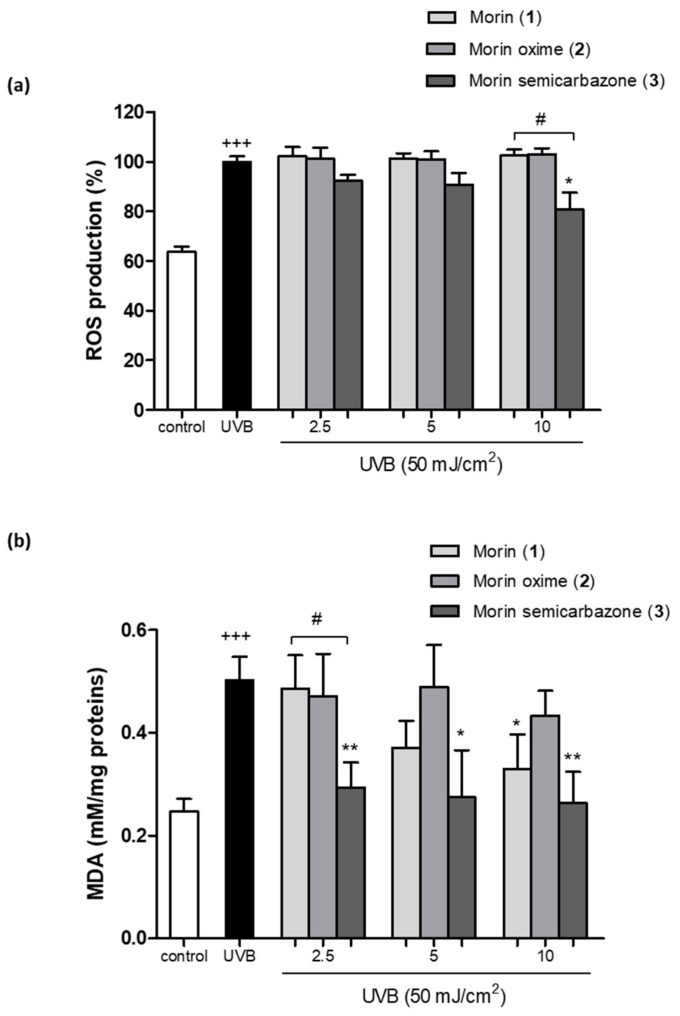
Evaluation of the ROS production and lipid peroxidation in UVB-irradiated HaCaT keratinocytes and pre-treated with morin (**1**) and its derivatives morin oxime (**2**) and morin semicarbazone (**3**). (**a**) Intracellular ROS production. Cells were pre-treated with the compounds at 2.5, 5, and 10 µM for 4 h and ROS levels were evaluated 45 min after UVB irradiation by the 2′,7′-dichlorodihydrofluorescein diacetate (DCF-DA) assay. Results are expressed as percentage with respect to UVB-exposed control cells. (**b**) MDA levels. Cells were pre-treated with the compounds at 2.5, 5, and 10 µM for 4 h and MDA levels were evaluated 24 h after UVB irradiation by thiobarbituric acid reactive substance (TBARS) assay. Results are represented as µM of MDA/mg protein. The bars represent the mean ± SEM (*n* = 6). The mean was found to be significantly different compared to the control cells (+++ *p* < 0.001; Mann–Whitney U-test). The mean was found to be significantly different compared to UVB-irradiated cells (* *p* < 0.05 and ** *p* < 0.01; Kruskal–Wallis test followed by Dunn’s multiple comparison test). The mean was found to be significantly different compared to compound **1** at the respective concentration (# *p* < 0.05; Mann–Whitney U-test).

In a first set of experiments, the scavenging capacity of **1** and the two semisynthetic derivatives (**2** and **3**) was evaluated by using the ABTS method. In this assay, the ABTS·+ radical was exposed to different concentrations of these phenolics (3.12–200 μM) to test their ability to donate electrons to this radical (Figure 3a). The effective concentration 50% (EC50) was calculated, and data were expressed as Trolox equivalent antioxidant capacity (TEAC), which is calculated as EC50 sample/EC50 Trolox (Figure 3b). The control reference compound Trolox exhibited an EC50 of 20.8 µM. Interestingly, the compounds **1**, **2**, and **3** exhibited a greater antioxidant capacity than that of Trolox, showing an EC50 of 10.8, 13.7, and 12.3 µM, respectively. The TEAC for **1**, **2**, and **3** showed 0.5, 0.7, and 0.6 equivalents of Trolox, which means that it is necessary to increase **1** 0.5-fold, **2** 0.7-fold, and **3** 0.6-fold to obtain a comparable effect to that of Trolox. In addition, the antioxidant activity was further evaluated on UVB-irradiated HaCaT by using the DCFH-DA technique (Figure 4a). The findings showed that UVB significantly increased intracellular ROS generation when compared with non-irradiated keratinocytes. Pre-treatment of cells with the compounds **1** or **2** did not induce significant changes in intracellular ROS levels. However, the derivative **3** reduced ROS production at all assayed concentrations in comparison with UVB-irradiated HaCaT, showing significance at the highest dose (10 µM). Interestingly, at 10 µM the compound **3** statistically reduced ROS levels by around 20% compared to the parent compound.

### 3.5. Effect of Morin (***1***) and Derivatives (***2*** and ***3***) Preventing Lipid Peroxidation

Lipid peroxides and their decomposition byproducts, including MDA, have the potential to modulate various crucial functions for maintaining cellular and organ balance, either directly or indirectly. Consequently, elevated membrane lipid peroxidation can trigger immune and inflammatory responses, induce gene expression and cell growth, or initiate apoptosis [24]. In this study, it was confirmed that UVB exposure significantly increased the levels of MDA, the main product of lipid peroxidation, in comparison to the non-irradiated cells (Figure 4b). Pre-treatment with **1** significantly reduced MDA levels only at the highest concentration (10 µM). As regards nitrogenated derivatives, no significant changes were observed after preincubation with the compound **2**. Interestingly, the compound **3** inhibited MDA formation at all tested concentrations (2.5, 5, and 10 µM) after UVB exposure. Moreover, MDA levels were reduced by around 30% by derivative **3** at 2.5 µM in comparison to **1**. This result showed it was necessary to have 4-fold more of compound **1** than **3** to observe a similar effect, this derivative exhibiting the highest lipid peroxidation inhibition activity.

### 3.6. Anti-Inflammatory Activity in UVB-Exposed HaCaT Cells

**Figure 5:**
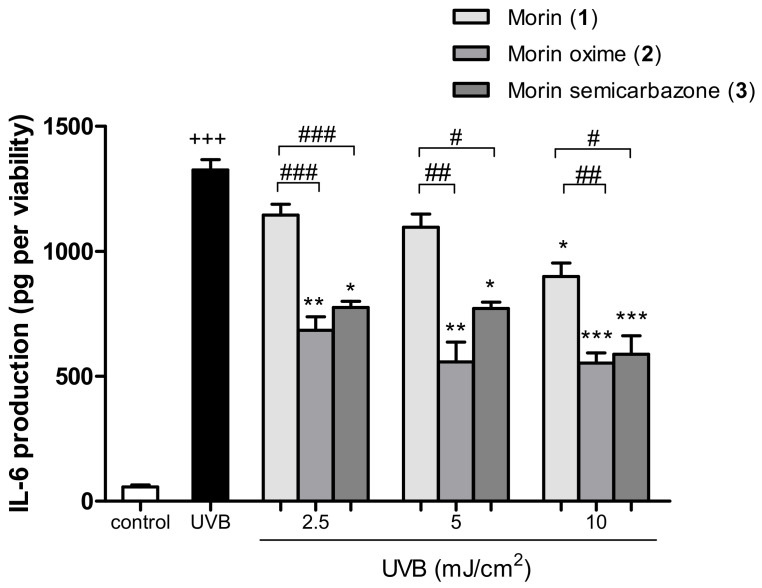
Anti-inflammatory effect of morin (**1**) and the two semisynthetic derivatives (**2** and **3**) on UVB-exposed HaCaT keratinocytes. The production of IL-6 in UVB-irradiated HaCaT cells and pre-treated with compounds **1**, **2**, and **3** (2.5, 5, and 10 µM) for 4 h was assessed using an ELISA kit assay (Diaclone GEN-PROBE, Besançon, France). Results are expressed as IL6 production (pg) and refer to the percentage of viable cells; and bars represent the mean ± SEM of six independent experiments (*n* = 6). The mean value was found to be significantly different compared to the control group (+++ *p* < 0.001; Studen’ *t*-test). The mean value was found to be significantly different compared to UVB-irradiated cells (* *p* < 0.05, ** *p* < 0.01 and *** *p* < 0.001; Kruskal–Wallis test followed by Dunn’s multiple comparison test). The mean was found to be significantly different compared to compound **1** at the respective concentrations (# *p* < 0.05, ## *p* < 0.01 and ### *p* < 0.001; Mann–Whitney U-test).

IL-6 is a pro-inflammatory cytokine involved in skin inflammatory responses, and it is widely recognized that UVB irradiation contributes to its release. IL-6 levels were measured 24 h after irradiation, confirming that UVB exposure significantly increased this cytokine release. Pre-treatment with **1** resulted in a significant decrease in IL-6 levels only at the highest concentration. However, the derivatives **2** and **3** significantly reduced IL-6 levels when HaCaT cells were pre-treated with all three tested concentrations (2.5, 5, and 10 µM) in comparison to UVB-irradiated cells alone (Figure 5). Interestingly, compounds **2** and **3** exhibited a more pronounced anti-inflammatory activity than that of the parent compound at all tested concentrations. These findings revealed it was necessary to have over 4-fold more of **1** than of 2.5 µM **2** and **3** to show a similar IL-6 production.

## 4. Discussion

Numerous advancements and research approaches are currently in progress within the fields of drug technology and chemistry. These efforts aim to enhance the effectiveness of drugs in terms of their therapeutic properties and to preserve their molecular integrity [25]. Literature has proposed that the bonding of compounds with bile acids has a favorable impact on photoprotection. This is evident as their sun-blocking capabilities were found to be on par or even superior to those of the unbound azastilbene. This observation implies that the non-steroidal component likely exerts a substantial influence on the ultimate photoprotection efficacy when these molecules are used as exclusive UV filters in the assessed products [26]. Expanding on previous investigations into chemical derivatization, the current study aims to chemically modify **1** to yield two derivatives with Schiff base structures, with the objective of enhancing its light absorption capability, particularly in the UV–Vis range, and its photoprotective effects. Conjugations with Schiff base chemical groups have been reported to improve antioxidant, antibacterial, or anticancer activities of the parent compounds [27,28,29]. In the present study, the carbonyl moiety from **1** favored the Schiff base reaction as expected [30]. The formation of compound **2** was revealed by key spectroscopic variations, including the observed IR vibrational band at 1654 cm^−1^ related to the C=N moiety [31], the slightly modified chemical shifts from morin skeleton after the reaction process (see experimental section) at ^1^H NMR, as well as the C-4 signal at ^13^C NMR. In a similar reasoning, the formation of compound **3** was deduced by the observed IR vibrational band at 1651 and 1608 cm^−1^ attributed to carbonyl groups from semicarbazone moiety. For this compound, the typical C=O resonance at ^13^C NMR from semicarbazone moiety [32] at δ 160.2 overlapped with the C-2 signal; however, the significant intensity of this signal was a crucial experimental finding, suggesting the formation of derivative **3**.

In terms of photoprotection, it can be inferred that an equivalent quantity of **2** or **3** is more effective than an equivalent amount of morin concerning absorption in the UV–Vis range, as evidenced by the absorbance peaks and molar extinction coefficients. This leads to the requirement of lower concentrations of these derivatives to achieve UV–Vis absorption values similar to those attained with **1**. From a chemical perspective, these modifications to **1** resulted in an enhanced capacity to absorb UV–Vis light, thereby improving and/or preserving the photoprotective activity of **1** itself, since oxime moiety for **2** or semicarbazone motif for **3** promote an enhancement on the conjugated system of the molecule involving an increment of the *π*-π* as well as n-π* transition possibilities during h*ν* excitation.

It is well-known that exposure of keratinocytes to UVB radiation leads to an increase in ROS levels and subsequent lipid peroxidation, resulting in photoaging and an increase in the risk of skin cancer [33]. Several lines of evidence have demonstrated that the use of antioxidants represents an effective strategy in photoprotection due to their capacity to protect cells from free radical generation and, consequently, mitigate skin cell damage. Therefore, because of their antioxidant properties, polyphenols have been used in skin care products to protect skin against UV-induced oxidative damage. In this regard, previous authors have demonstrated that morin protected cells against the damage induced for both UVB and UVA radiation in keratinocyte stem cells and in dermal fibroblasts, respectively [34,35]. In addition, morin loaded in nanoparticles or niosomes has demonstrated antioxidant and anti-aging activities, acting as a free radical scavenging compound [12,16]. Consistent with these previous studies, our results demonstrated that **1** protected cells from UVB irradiation-induced cell death and prevented lipid peroxidation. Regarding the chemical modifications of **1**, the incorporation of Schiff bases, in addition to enhancing its absorption capacity in the UV–Vis range, improved the photoprotective activity of the parent compound. Specifically, the derivative **3** demonstrated the strongest antioxidant activity through reduced UVB-induced ROS generation and, subsequently, induced a marked reduction in MDA levels, being the most antioxidant compound. Thereby, semicarbazone moiety resulted in an effective pharmacophore that improves the antioxidant potential in cells [36].

The excessive ROS levels after UVB exposure also induce the release of pro-inflammatory cytokines by keratinocytes, such as IL-6, into the cell environment, which plays a crucial role in UVB irradiation-induced photoaging and skin inflammation. Previous in vitro studies have reported that **1** inhibited UVB-induced production of inflammatory cytokines, such as TNF-α, IL-1β, and IL-6 in human keratinocyte stem cells [34]. Our findings revealed that **1** was only able to induce a marked reduction in IL-6 levels at the highest concentration assayed. Interestingly, the anti-inflammatory efficacy of the two morin derivatives **2** and **3** was better than that of **1** since both compounds significantly reduced IL-6 production at the three concentrations used in this study.

Given that incomplete photoprotection can arise from various factors, including the amount or frequency of application, as well as the photodegradation of sunscreens due to factors such as heat, time, and other environmental elements, there is a pressing need for improved photoprotective measures. This may involve a double focus on education and the development of more stable formulas [5]. In this regard, the incorporation of new compounds into sunscreen formulations could provide complementary attributes to the physical and chemical filters, enabling a more comprehensive cellular protection, and ensuring antioxidant or anti-inflammatory activities [2]. Our findings suggest an advancement through the optimization of natural active ingredients and the potential inclusion of these in topical sunscreens. Notably, morin-Schiff base derivatives exhibit dual functionalities, encompassing both photoabsorption capacity and biological effects. Furthermore, the use of morin derivatives as adjuncts in photoprotection could potentially prevent the development of cancerous lesions, mitigate the exacerbation of photodermatoses and reduce premature skin aging induced by solar radiation exposure.

## 5. Conclusions

Overall, this research implies progress in the exploration of natural compounds with effective photoprotective properties. The incorporation of Schiff bases to **1** so as to synthetize the two derivatives (**2** and **3**) has shown for the first time a promising effect of enhancing photoprotection and preventing UVB-induced skin damage, which is potentially linked to mitigating the development of premature aging and skin cancer. Therefore, these findings suggest the use of these morin-Schiff base derivatives as natural sunscreen ingredients and open new avenues for the development of advanced photoprotective formulations. However, further studies are required to fully elucidate the underlying mechanisms by which these bioactive compounds exert their photoprotective effects and, more specifically, the potential influence of Schiff bases on DNA alterations induced by solar radiation.

## 6. Patents

A patent resulting from the work reported in this manuscript has been presented in the Spanish Patent and Trademark Office (number P202330632).

## Data Availability

Data are contained within the article and Supplementary Materials.

## Supplementary Materials

The following supporting information can be downloaded at https://www.mdpi.com/article/10.3390/antiox13010134/s1, Figure S1: ^1^H NMR spectrum of morin oxime (**2**), at 400 MHz, in DMSO-d6; Figure S2: ^13^C NMR spectrum of morin oxime (**2**), at 100 MHz, in DMSO-d6; Figure S3: ^1^H NMR spectrum of morin semicarbazone (**3**), at 400 MHz, in DMSO-d6; Figure S4: ^13^C NMR spectrum of morin semicarbazone (**3**), at 100 MHz, in DMSO-d6; Table S1: Molar absorption coefficient values of morin (**1**) and its derivatives morin oxime (**2**) and morin semicarbazone (**3**).
