# Supplementary material for: Photoprotective Effects of Two New Morin-Schiff Base Derivatives on UVB-Irradiated HaCaT Cells
Source: Antioxidants (Basel). 2024 Jan 22;13(1):134. doi: 10.3390/antiox13010134 (PMC10813227; doi:10.3390/antiox13010134)
Supplement: Supplementary file 1 [file antioxidants-13-00134-s001.zip › antioxidants-2804197-supplementary.pdf]

antioxidants

MDPI

Supplementary Material

Figures:

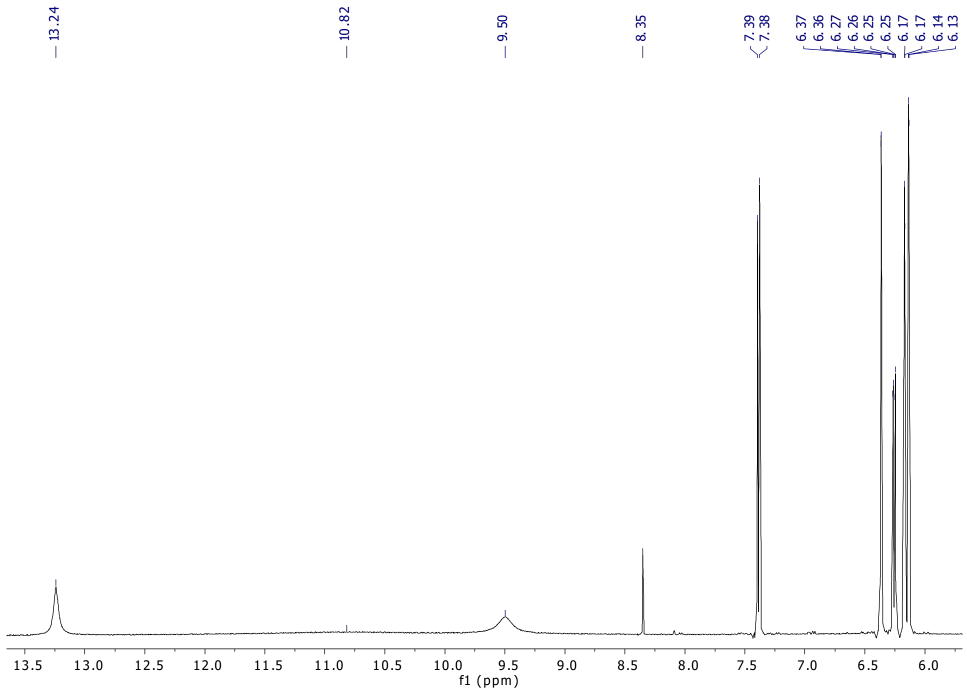

Figure S1. 1H NMR spectrum of morin oxime (2), at 400 MHz, in DMSO-d6;.

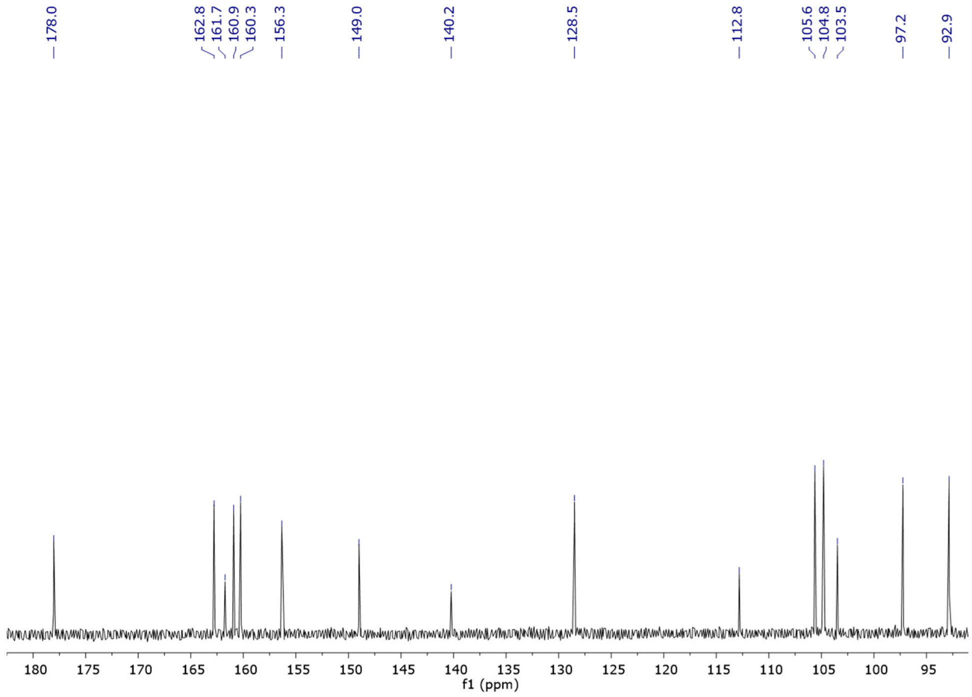

Figure S2. 13C NMR spectrum of morin oxime (2), at 100 MHz, in DMSO-d6;.

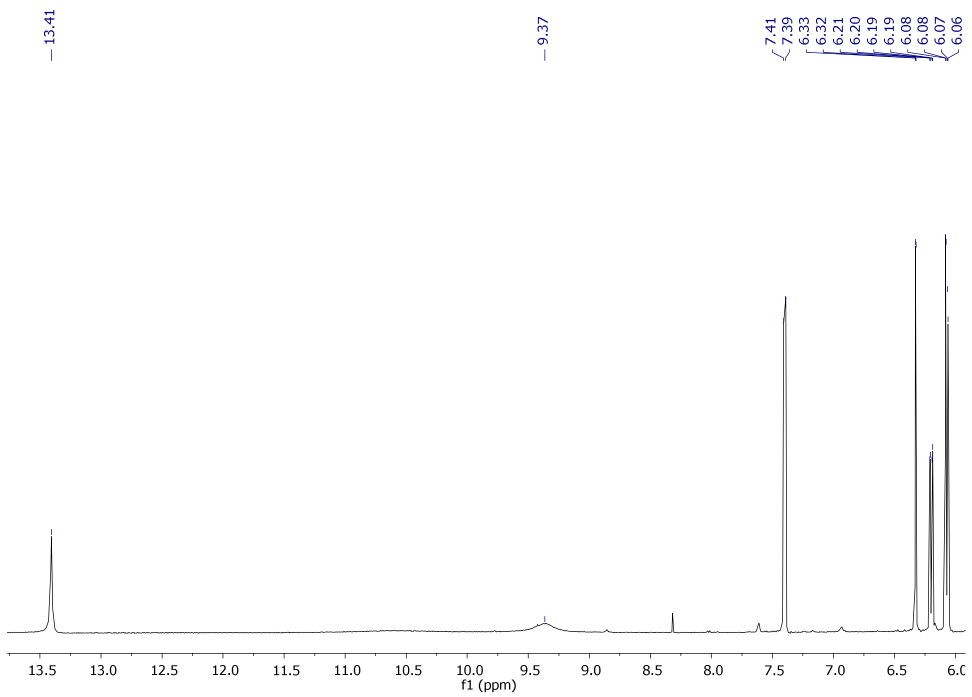

Figure S3. 1H NMR spectrum of morin semicarbazone (3), at 400 MHz, in DMSO-d6;.

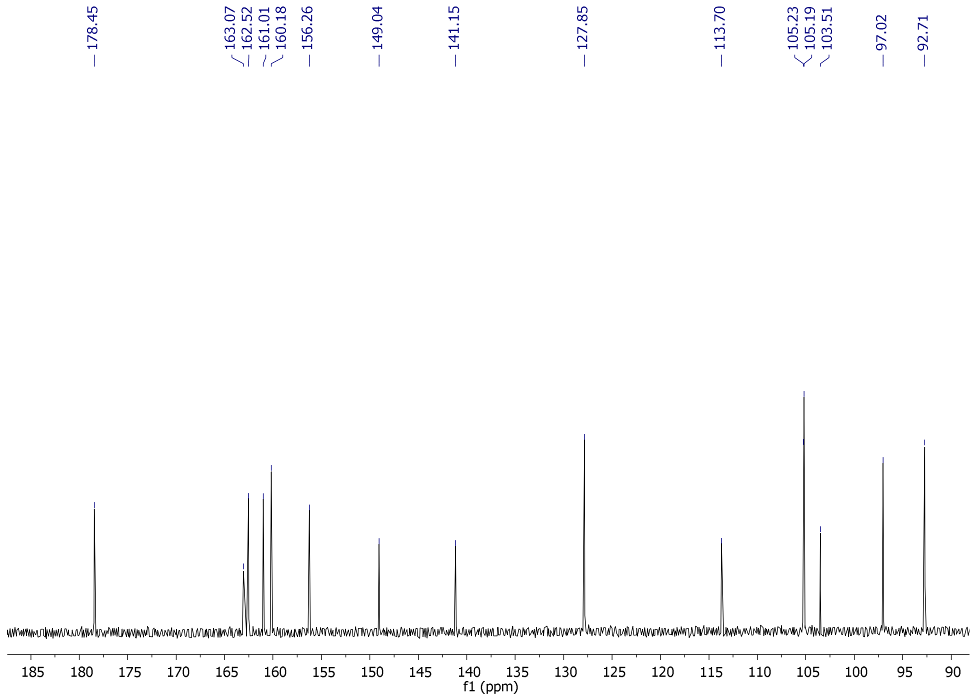

Figure S4. 13C NMR spectrum of morin semicarbazone (3), at 100 MHz, in DMSO-d6;.

Table:

Table S1. Molar absorption coefficient values of morin (1) and its derivatives morin oxime (2) and morin semicarbazone (3).

**λ (nm)**	**Molar absorption coefficient (*ε*)**		
	**1**	**2**	**3**
210	40426	35090	25453
265	25450	16756	16664
390	29976	13198	16549
